# Genetic Consequences of Antiviral Therapy on HIV-1

**DOI:** 10.1155/2015/395826

**Published:** 2015-06-10

**Authors:** Miguel Arenas

**Affiliations:** ^1^Institute of Molecular Pathology and Immunology of the University of Porto (IPATIMUP), 4200-465 Porto, Portugal; ^2^Centre for Molecular Biology “Severo Ochoa”, Consejo Superior de Investigaciones Científicas (CSIC), 28049 Madrid, Spain

## Abstract

A variety of enzyme inhibitors have been developed in combating HIV-1, however the fast evolutionary rate of this virus commonly leads to the emergence of resistance mutations that finally allows the mutant virus to survive. This review explores the main genetic consequences of HIV-1 molecular evolution during antiviral therapies, including the viral genetic diversity and molecular adaptation. The role of recombination in the generation of drug resistance is also analyzed. Besides the investigation and discussion of published works, an evolutionary analysis of protease-coding genes collected from patients before and after treatment with different protease inhibitors was included to validate previous studies. Finally, the review discusses the importance of considering genetic consequences of antiviral therapies in models of HIV-1 evolution that could improve current genotypic resistance testing and treatments design.

## 1. Introduction

According to UNAIDS, the Joint United Nations Programme on HIV/AIDS World Health Organization, a total of 35.3 [32.2–38.8] million people worldwide were living with HIV-1 in 2012, indicating a ~15% increase of infected people from 2001 [1]. A total of 2.3 [1.9–2.7] million were newly infected during 2012, showing a 33% decline of new infections from 2001 with 3.4 [3.1–3.7] million. Indeed, the number of AIDS deaths declined from 2.3 [2.1–2.6] million in 2005 to 1.6 [1.4–1.9] million in 2012 [1]. An important cause for such a death decline is the antiretroviral therapy, usually referred to as highly active antiretroviral therapy (HAART). In 2012, a total of 9.7 million people from low/middle-income countries received HAART and the UNAIDS expects to reach 15 million people receiving HAART by 2015 [1]. Nevertheless, in 2013 only 34% of people infected with HIV in low/middle-income countries (28.6 million) could receive therapy [1]. Therefore, there are still important regional differences that should be solved [2, 3]. On the other hand, the development of an effective HIV vaccine is still under progress with a number of failures [4] because of the high rate of evolution of HIV-1 [5, 6]. As a consequence, up to date the only treatment for HIV-1 is the antiretroviral drug therapy.

HAART have largely delayed the onset of AIDS-related illness and death [1] although they cannot eradicate the virus mainly due to latent viral reservoirs [7]. In addition, drug resistance mutations can reduce the activity of the therapy [8, 9]. Drug resistance mutations probably emerge because HIV evolves rapidly, with high mutation and recombination rates and under rapid population dynamics [10]. Of course, then natural and drug-induced selection can eliminate most of viral variants [11]. The surviving variants (8–20%) present drug resistance mutations, which allows recovering fitness and replication capacity [8, 12]. Interestingly, different inhibitors can generate different selective pressures that induce the fixation of different resistance mutations in the viral population but also different resistance mutations may affect different inhibitors in a different fashion. This suggests the simultaneous use of more than one inhibitor that could cover a wider range of mutations [13], although this strategy may fall into similar resistance (cross-resistance) and lack of synergy [14, 15].

A potential strategy to deal with the problem of resistance mutations could be the consideration of the molecular evolution of the virus [16, 17] into the inhibitor design. For example, inhibitors that account for molecular evolutionary processes of the virus could eliminate viral variants that could be predicted beforehand. Actually, this promising strategy is commonly applied to HIV-1 vaccines design through the use of centralized (consensus, center-of-tree or ancestral) genes that can induce immune responses (reviewed in [17]). Such centralized sequences could consider the immunogenetic particularities of the diverse circulating variants in the target population [18, 19]. However, although these centralized vaccines generated promising antibody responses, they were only partially effective in covering the large HIV-1 genetic diversity. Perhaps this could be derived from the application of not enough realistic models of HIV-1 evolution as suggested in [17]; see also [20]. Knowledge on HIV-1 molecular evolution can also be used to develop realistic models of evolution [21, 22] that can be applied for additional purposes such as the prediction of resistance mutations [23] or the evolutionary reply of the viral population, genotypic resistance testing [24, 25].

This study explores the genetic consequences of antiviral therapy on HIV-1. First, it analyzes the influences of antiviral therapies on the viral genetic diversity, including the particular roles of the substitution and recombination processes in the generation of drug resistance. Then, the molecular signatures of selective pressures derived from antiviral therapies are evaluated. A brief evolutionary analysis of the influence of different protease (PR) inhibitors (PIs) on the PR-coding region was performed to evaluate previous works and to provide an illustrative example. The application of the genetic consequences derived from antiviral therapies in the development of new empirical substitution models that could be used for purposes such as genotypic resistance testing and treatments design is also discussed.

## 2. Genetic Diversity Generated during HIV-1 Antiviral Therapy

Interestingly, the effects of HIV-1 antiviral drugs on the viral genetic diversity depend on the evolutionary level under study. It differs from overall diversity of circulating strains in the viral population to local nucleotide diversity of particular viral genes.

The antiviral therapy can reduce the global viral genetic diversity in the population due to the selection of viral strains [26–28]. This phenomenon can be interpreted as a classical population range contraction and habitat fragmentation that commonly tend to decrease genetic diversity [29, 30]. Actually, a variety of population genetics analysis of HIV-1 showed the existence of severe population bottlenecks (loss of viral load) and loss of virus fitness during drug regimens [28, 31, 32].

In contrast, the survival strains may present drug resistance mutations [33] that often increase genetic diversity of the protein-coding genes of the target proteins [12, 34, 35]. Wu et al. [36] found that patients treated with several PIs presented 3 times more protease mutations than untreated patients. These findings are also observed in the computational analysis presented in the last section of this paper where most of PIs promoted higher levels of nucleotide diversity in the PR-coding gene. Interestingly, pairs and clusters of correlated resistance mutations (coevolution) were significantly more abundant in treated patients [36]. Consequently, the increased diversity does not follow a random process, instead the new mutations present residue-residue interactions from direct association with viral protein inhibitors [23]. Increased genetic diversity can also be observed under treatment with other antiviral drugs such as reverse transcriptase (RT) inhibitors [34, 37–39] and integrase (IN) inhibitors [40–43], although the increased diversity under the latter drug class is mainly based on secondary resistance mutations [40–43]. Indeed, combinations of different drug classes (acting on different HIV-1 proteins) can generate synergistic inhibition [44] but the overall presence of synergy on genetic diversity remains to be explored, although some mutations in the* Env* region have already been associated with resistance to entry inhibitors that affect other viral genes [9]. Overall, at this level, the molecular mechanisms by which the virus can evade treatments seem directly related with the virus's ability to generate genetic diversity in a particular environment. Thus, this increased genetic diversity could be driven by strong selective pressures (discussed later).

## 3. The Role of Viral Recombination during HIV-1 Antiviral Therapy

Recombination constitutes a fundamental evolutionary force in HIV generating new viral strains, increasing viral diversity, and facilitating adaptation [45–47]. Indeed, ignored recombination can bias the inference of a variety of evolutionary processes and parameters (i.e., it can increase the number of false positively selected sites [48, 49] or generate incorrect phylogenetic tree and ancestral sequence reconstructions [50, 51]). Therefore recombination should be taken into account for analyzing and understanding HIV-1 evolution.

The role of recombination on the emergence of drug resistance mutations is not yet clear and it can be difficult to assess because other processes may also influence its evolutionary consequences (i.e., cellular superinfection [52–54], random genetic drift, and viral population size [55, 56] or fitness selection of the newly generated viral forms [57, 58]) and because the detection of recombination can be problematic under low levels of nucleotide diversity [59]. Contradictory effects of recombination during HIV-1 antiviral therapy can be found in the literature.

As one would expect beforehand, several studies showed that recombination is crucial to generate drug resistance. A computer simulations study suggested that recombination might favor the generation of drug resistance [60]. In addition, HIV-1 strains derived from recombination events presented resistance mutations [61, 62].

On the contrary, Archer et al. [63] showed that despite the wide diversity of recombinant forms in HIV populations, only a minority of recombination events are of significance to the evolution of the virus. Counterintuitively, it has also been demonstrated that recombination can slow down the generation of multi-drug-resistant strains during therapy [52] and it may be suppressed by selection for resistance to PIs [64].

It seems that the initial genetic barrier caused by recombination (most of recombinant forms could present low fitness) could reduce the fitness of the viral population during the therapy but in case a recombinant form is selected, resistance mutations could be better able to persist in the viral population [54] and speed up adaptation (the Fisher-Muller effect) [65]. In any case, these opposite findings suggest that more sophisticated analyses should be performed to determine the influence of recombination on the emergence of drug resistance mutations, as suggested by Shi et al. [61].

## 4. Selective Pressures Induced by HIV-1 Antiviral Therapy

Antiviral therapy may cause important selective pressures on viral populations [12, 66]. In particular, severe fitness losses can be derived from antiviral treatments until the emergence of beneficial mutations that allow restoring the vital replication capacity [12]. Thus, resistance to viral inhibitors can drive the fixation of favorable variants [23, 67].

The overall response to antiviral drugs presented an excess of nonsynonymous substitutions [23, 68] (which was also found in the analysis presented in the following section). For example, Wu et al. [36] found that an antiviral therapy can induce diversifying selection in nearly one-half of PR sites. It is widely known that positively selected sites (PSSs) are often located in the protein surface, whereas conserved or negatively selected sites (NSSs) are commonly observed in the protein core in order to conserve the protein function [69]. However, the molecular adaptation induced by antiviral therapies does not present such a scenario. Poon et al. [23] found that the distribution of nonsynonymous substitutions along the gene is shaped by selection to PI resistance. Moreover, antiviral therapies promote complex drug-specific residue-residue interaction networks [23, 70, 71] that can drive the coevolution of primary and secondary resistance mutations [8, 23].

## 5. Genetic Impact of Diverse PIs on HIV-1 PR-Coding Genes: A Computational Study

The HIV-1 PR is one of the most used drug targets for combating HIV with a number of chemically diverse inhibitors that have already been tested [72, 73]. This section includes a computational analysis of nucleotide diversity and molecular adaptation of the PR-coding gene evolution under different PIs.

### 5.1. Sample Collection

Samples of coding DNA sequences that encode the HIV-1 PR (*Pol* region, subtype B) were collected from the Stanford HIV Drug Resistance Database [74, 75]. Subtype B was used because most (~99%) of datasets available in the database belong to this subtype and there is not enough data to analyze other subtypes. For each HIV-1 patient, a clonal sequence was collected under no-treatment and another one was collected after a particular treatment based on a single PI or a PIs combination. According to the detailed information provided by the database [74, 75], the patients did not receive other treatments. Therefore, to study each treatment (hereafter, evolutionary scenario) two datasets (pool of sequences before and after treatment) were obtained. In particular, for each evolutionary scenario, a dataset includes coding sequences collected before a given treatment and the other dataset includes coding sequences collected after such a treatment, and both datasets come from the same patients. As suggested by Kosakovsky Pond and Frost [76], scenarios with sample size lower than 10 were not considered to avoid lack of power in the evolutionary analysis (datasets with higher sample size can generate accurate estimates of genetic diversity and nonsynonymous to synonymous substitution rates ratio (*dN/dS*) [76]; see also [3, 77]). A total of 13 evolutionary scenarios, all the currently available scenarios from the database, were analyzed. Namely, a “control” scenario (no-treatment in both datasets, scenario 1, 1011 patients) and scenarios with the following treatments:* amprenavir* (APV, scenario 2, 15 patients),* atazanavir* (ATV, scenario 3, 23 patients),* indinavir* (IDV, scenario 4, 77 patients),* lopinavir* (LPV, scenario 5, 34 patients),* nelfinavir* (NFV, scenario 6, 317 patients),* ritonavir* (RTV, scenario 7, 24 patients),* saquinavir* (SQV, scenario 8, 35 patients), and the PI combinations: IDV + RTV (scenario 9, 10 patients), RTV + SQV (scenario 10, 11 patients), and IDV + RTV + SQV (scenario 11, 11 patients). Two additional scenarios were also studied where patients treated with IDV are then treated with IDV + NFV (scenario 12, 13 patients) or IDV + RTV (scenario 13, 16 patients).

### 5.2. Analysis of Genetic Diversity and Recombination

Several genetic statistics were applied to study the influence of PIs on the genetic diversity of the PR-coding gene. (i) The overall sequences divergence was computed with* MEGA 6.0* [78]. (ii) Nucleotide diversity (*π*) was estimated by using the pairwise nucleotide differences per site [79]. These metrics considered indels as missing data. (iii) The genetic distance between the two datasets of each evolutionary scenario was computed by the Kullback-Leibler (KL) divergence [80] and considering indels as missing data and as an additional state. This distance provides a comparative analysis of nucleotide diversity distributions across sites between two datasets [81].

**Figure 1:**
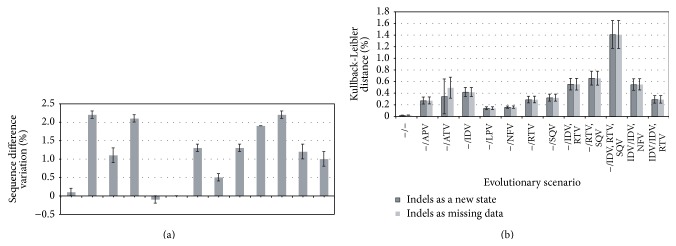
Overall sequence diversity variation and Kullback-Leibler divergence. (a) Variation of overall sequence difference between the two datasets of each evolutionary scenario (*d*_after  treatment_ − *d*_before  treatment_). Indels are considered as missing data. Error bars indicate standard error. Reference values are shown in Table S1 (Supplementary Material). (b): Kullback-Leibler distance, nucleotide diversity distribution, between the two datasets of each evolutionary scenario. Dark grey bars consider indels as a new state whereas clear grey bars consider indels as missing data. Error bars indicate standard error across sites. “−” indicates naïve-treatment patients.

**Figure 2:**
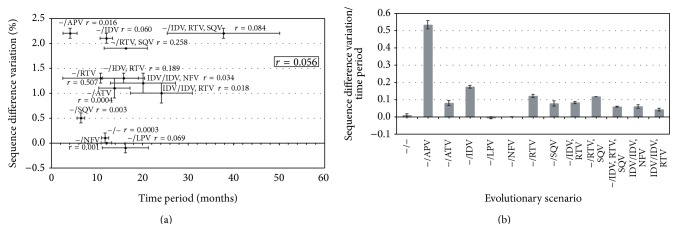
Sequence difference variation as a function of time interval between samples. (a) Variation of the overall sequence difference between the two datasets of each evolutionary scenario (*d*_after  treatment_ − *d*_before  treatment_) is represented in the “*y*-axis” (mean and standard error). The time period between both samples (*t*_after  treatment_ − *t*_before  treatment_) is represented in the “*x*-axis” (mean and standard error from all patients of the scenario). The correlation coefficient between both parameters among all the scenarios is *r* = 0.056, suggesting absence of correlation. Correlation coefficients within each evolutionary scenario (among patients) between these parameters are also shown in the plot and ranges from 0.0003 to 0.507, although most of them are under 0.1. (b) Genetic diversity gradient divided by the corresponding time period. Error bars indicate standard error. “−” indicates naïve-treatment patients. Reference values are shown in Table S1 (Supplementary Material).

Briefly, the results show that almost all PIs lead to higher levels of sequences divergence, pairwise nucleotide diversity, and nucleotide diversity distribution across sites. Except for LPV and NFV, all PIs increased the overall difference between sequences (Figure 1(a)). Similar results are derived from the estimates of nucleotide diversity although here only LPV presented low levels of nucleotide diversity variation (see Figure S1 in Supplementary Material available online at http://dx.doi.org/10.1155/2015/395826). The highest levels of diversity were generated from treatment with APV, IDV, and, especially, PIs combinations. However, the increase of diversity could be caused by the emergence of resistance mutations but also by mutations derived from the natural evolution of the gene. Therefore, it is interesting to evaluate the correlation between the variation of diversity and the corresponding time period between samples.  Figure 2(a) suggests that there is no correlation between these parameters, which is supported by a low correlation coefficient (*r* = 0.056). For example, the control dataset (no-treatment) does not present increase of diversity despite its long time period (11 months), whereas treatment with APV generated one of the highest levels of diversity in only 4 months (Figure 2). In addition, correlation coefficients within scenarios (among patients from a particular scenario) were also very low, most of them under 0.1 (Figure 2(a)). A normalization dividing the genetic diversity gradient by the time period between samples also indicated the increase of genetic diversity with most of PIs (Figure 2(b)). However, the normalization must be carefully interpreted because a longer time period does not necessarily lead to more diversity [82], which is actually indicated by the described lack of correlation.

The analysis of nucleotide diversity distribution across sites between the two alignments showed similar findings for most of PIs (Figure 1(b)). Notice that this nucleotide distribution can be more influenced by several mutations at specific positions [80] and therefore this statistic might be more sensible to detect resistance mutations. The results show an influence of all PIs on the nucleotide diversity distribution (Figure 1(b)), although this influence varies among inhibitors. Again, the long KL distance derived from drug combination therapies is remarkable.

Absence of recombination breakpoints was found with the single breakpoint position (SBP) method [83], implemented in the* Hyphy* package [84], and with the recombination detection methods implemented in the* RDP* framework [85].

### 5.3. Signatures of Molecular Adaptation

The best-fit model of DNA substitution was selected with* jModelTest* [86] under the Bayesian information criterion (BIC), as suggested by [87]. Then, maximum likelihood (ML) phylogenetic trees were inferred with* PhyML* [88] under the corresponding substitution model. These trees were used to perform the molecular adaptation inferences. Estimates of* dN/dS* at both global (sequence) and local (codon) levels were performed with the Fixed Effects Likelihood (FEL) method [76] implemented in the* Hyphy* package. Notice that this ML-based method provides very accurate estimates [76] and it is commonly used in population genetics and virus evolution (e.g., [3]).

**Figure 3:**
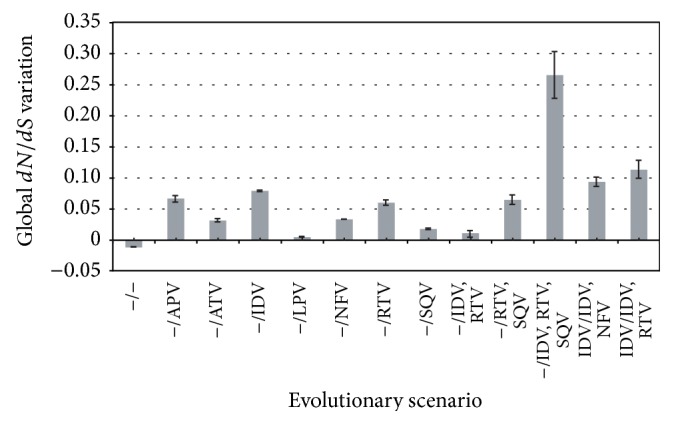
Global nonsynonymous to synonymous substitution rates ratio (*dN/dS*) variation. Variation of global* dN/dS* between the two datasets of each evolutionary scenario (*dN*/*dS*_after  treatment_ − *dN*/*dS*_before  treatment_). Error bars indicate 95% CI. “−” indicates naïve-treatment patients. Reference values are shown in Table S3 (Supplementary Material).

Figure 3 shows the variation of global* dN/dS* estimates from datasets collected before and after a treatment. All the PIs promoted increased estimates of* dN/dS*, especially when the treatment is based on PIs combination. By contrast, in absence of treatment the estimated* dN/dS* declined with time. At the local level, almost all the PIs promoted an increase of significant (*p* value < 0.05) PSSs along the PR-coding sequence (Figure S2 and Table S4, Supplementary Material). In general, a large number of NSSs were detected in all datasets (Table S4) without showing a clear relationship with the presence or absence of treatment. All these results are discussed in the next section.

## 6. Concluding Remarks

The fast population range contractions and fragmentation produced during the therapy can reduce the overall diversity of viral strains [29] and, by contrast, the emergence of resistance mutations caused from the rapid evolution of HIV allows preserving or increasing the levels of nucleotide diversity of viral protein-coding genes of the drug target. Indeed, resistance mutations can be rare, but also recurrent enough until they reach resistance, and can generate positive selection driving the fixation of favorable viral strains [23, 67]. At this level two opposite selective pressures seem to act. While most of sites evolve under strong purifying selection, probably caused by the host's immune system and the therapy, other sites evolve under diversifying selection, probably caused by the viral molecular adaptation to the new environment established by the therapy, and can present complex residue-residue interaction networks suggesting dependent evolution among sites [23, 70].

The evolutionary analysis included in this work supported such considerations. It also showed that different PIs can promote different influences on the genetic diversity of the viral PR-coding gene. For example, IDV and APV induced the highest levels of diversity (among treatments with a single PI) and PIs combination induced very high levels of genetic diversity, especially when 3 PIs are applied jointly. As noted, this increase of diversity can be related with the emergence of resistance mutations [33]. The reason why some inhibitors induce more diversity than others requires complex structural analysis of enzyme-inhibitor interactions, which is an important topic of research [89, 90]. On the other hand, absence of recombination breakpoints was found in the analyzed PR-coding genes. This could be caused by limitations to detect recombination under low genetic diversity levels [59] or just because recombination was not required to generate drug resistance. Indeed, recombination could occur in other genomic regions [63] (i.e., recombinants with breakpoints in* Gag* and* Pol* may present selection against [63, 91]). Concerning molecular adaptation, the results showed that the wild evolution of the virus presents an overall decrease of the global* dN/dS*, without PSSs and where most of sites evolved under significant purifying selection. This is probably caused by the sharp purifying selection induced by the host's immune system. On the other hand, PIs often promoted an overall increase of the global* dN/dS* (see [23, 68] and Figure 3), which is most of times accompanied by the emergence of significant PSSs along the gene (see [23] and Figure S2). As expected, the largest* dN/dS* increase occurs under treatments with PIs combination (see [23] and Figure 3). These signatures of molecular adaptation are related with the amount of genetic diversity induced by the PIs and indicate the primary importance of adaptation in the evolutionary process of the PR-coding gene under PIs. On the other hand, a large number of codons evolved under negative (purifying) selection, which indicates the presence of strong selective pressures, as noted probably caused by the host's immune system and the therapy.

Understanding molecular evolution of the virus can help us develop more realistic models of HIV evolution [23, 69, 71], correlate the disease progression with the evolution of the viral population [28, 92], and predict resistance (i.e., by genotypic-resistance testing [24, 25, 93, 94]) and common ancestry [18], or vaccine design [17–19]. Nevertheless, HIV-1 evolution is complex and other phenomena should also be taken into account as much as possible in the models, for example, different host's immune responses, clinical stage, HIV-1 compartmentalization [95], or infection with multiple viral variants, although the latter presents an overall low incidence [53, 96].

Since antiviral therapies affect genetic diversity of the virus by strong selective pressures, models of HIV-1 evolution should accommodate such effects in order to mimic these scenarios for purposes such as robust genotypic resistance testing and treatments design. Importantly, models of HIV-1 evolution should be as realistic as possible in order to provide accurate predictions. A possibility could be the consideration of a fitness landscape (e.g., [97]) to develop parametric models. However, the design and computation of a realistic fitness function are too convoluted due to complex processes that affect viral genetic diversity such as antiviral therapies (as noted in this paper). An easier, but less robust, alternative can be the development of scenario-specific empirical models. As shown in this paper, different therapies must be modeled with different models of evolution since different therapies can promote different genetic consequences in the virus. Much more research is needed (i.e., the consideration of associations between observed genotypes and phenotypic resistance in models of HIV-1 evolution) but my impression is that HIV-1 therapies will benefit from more consideration of evolutionary information.

## Supplementary Material

Figure S1. Nucleotide diversity variation.
Figure S2. Histogram on the distribution of the significant nonsynonymous substitutions (95% CI), induced by the studied PIs, along the PR coding gene.
Table S1. Estimates of overall mean distance with *MEGA*.
Table S2. Estimates of nucleotide genetic diversity.
Table S3. Estimates of molecular adaptation with *Hyphy*.
Table S4. Number and position of positively selected sites and negatively selected sites.
