# Supplementary material for: Genetic Consequences of Antiviral Therapy on HIV-1
Source: Comput Math Methods Med. 2015 Jun 10;2015:395826. doi: 10.1155/2015/395826 (PMC4478298; doi:10.1155/2015/395826)
Supplement: Supplementary file 1 — Figure S1. Nucleotide diversity variation. Figure S2. Histogram on the distribution of the significant nonsynonymous substitutions (95% CI), induced by the studied PIs, along the PR coding gene. Table S1. Estimates of overall mean distance with MEGA. Table S2. Estimates of nucleotide genetic diversity. Table S3. Estimates of molecular adaptation with Hyphy. Table S4. Number and position of positively selected sites and negatively selected sites. [file 395826.f1.pdf]

Supplementary Material

Genetic Consequences of Antiviral Therapy on HIV-1

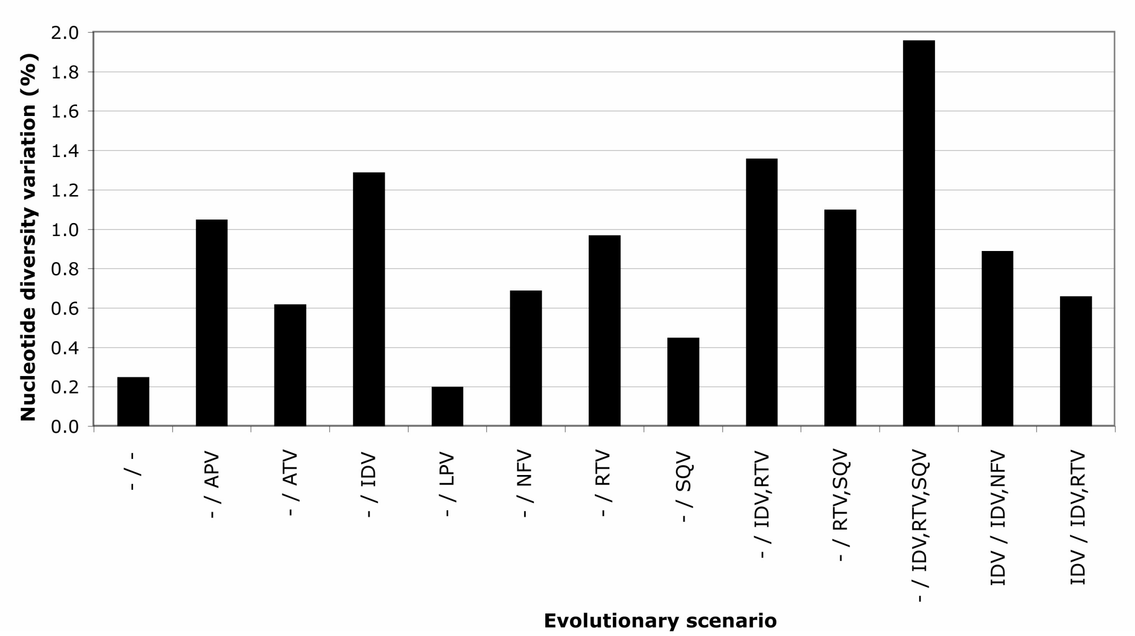

Figure S1. Nucleotide diversity variation. Variation of nucleotide diversity (pairwise nucleotide differences per site π) between the two datasets of each evolutionary scenario ($\pi_{\text{after treatment}}$ - $\pi_{\text{before treatment}}$). "-" indicates naïve-treatment patients. Reference values are shown in Table S2.

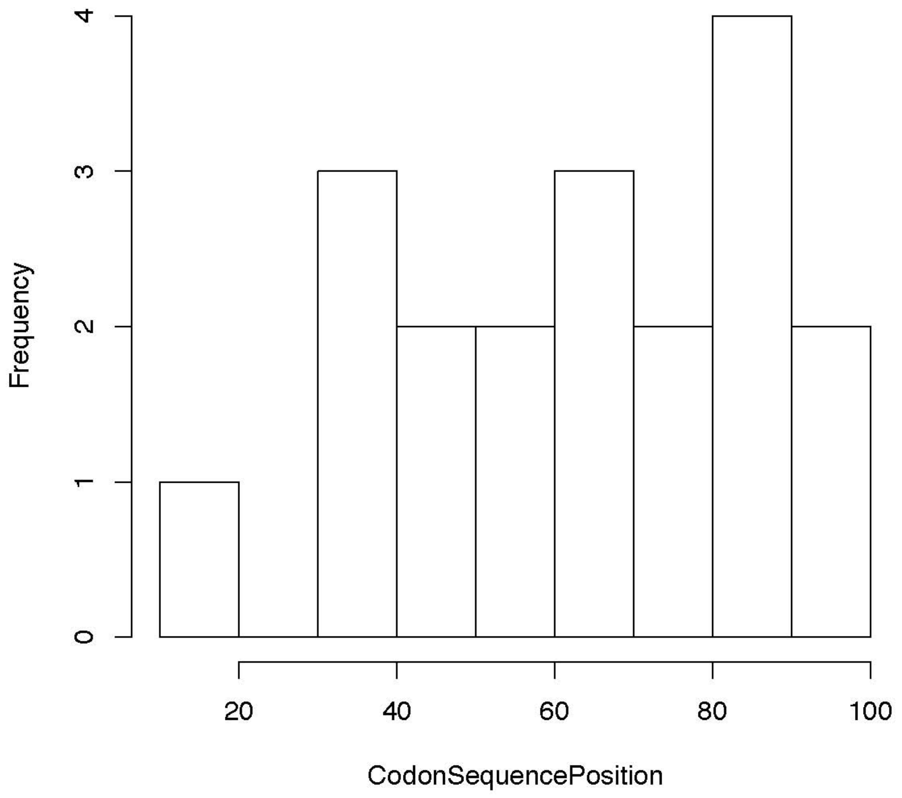

Figure S2. Histogram on the distribution of the significant nonsynonymous substitutions (95% CI), induced by the studied PIs, along the PR coding gene. Further details are included in Table S4.

Table S1. Estimates of overall mean distance with *MEGA*.

Dataset	Overall mean distance	Standard error
- / - (before treatment)	0.001	0.000
- / - (after treatment)	0.002	0.001
- / APV (before treatment)	0.028	0.005
- / APV (after treatment)	0.050	0.006
- / ATV (before treatment)	0.034	0.005
- / ATV (after treatment)	0.045	0.007
- / IDV (before treatment)	0.029	0.005
- / IDV (after treatment)	0.050	0.006
- / LPV (before treatment)	0.052	0.007
- / LPV (after treatment)	0.051	0.008
- / NFV (before treatment)	0.003	0.001
- / NFV (after treatment)	0.003	0.001
- / RTV (before treatment)	0.038	0.006
- / RTV (after treatment)	0.051	0.007
- / SQV (before treatment)	0.047	0.007
- / SQV (after treatment)	0.052	0.006
- / IDV,RTV (before treatment)	0.059	0.010
- / IDV,RTV (after treatment)	0.072	0.009
- / RTV,SQV (before treatment)	0.055	0.010
- / RTV,SQV (after treatment)	0.074	0.010
- / IDV,RTV,SQV (before treatment)	0.050	0.007
- / IDV,RTV,SQV (after treatment)	0.072	0.008
IDV / IDV,NFV (before treatment)	0.057	0.008
IDV / IDV,NFV (after treatment)	0.069	0.010
IDV / IDV,RTV (before treatment)	0.073	0.008
IDV / IDV,RTV (after treatment)	0.083	0.010

Table S2. Estimates of nucleotide genetic diversity.

Dataset	π
- / - (before treatment)	0.0653
- / - (after treatment)	0.0678
- / APV (before treatment)	0.0376
- / APV (after treatment)	0.0481
- / ATV (before treatment)	0.0479
- / ATV (after treatment)	0.0541
- / IDV (before treatment)	0.0498
- / IDV (after treatment)	0.0627
- / LPV (before treatment)	0.0739
- / LPV (after treatment)	0.0759
- / NFV (before treatment)	0.0429
- / NFV (after treatment)	0.0498
- / RTV (before treatment)	0.0496
- / RTV (after treatment)	0.0593
- / SQV (before treatment)	0.0474
- / SQV (after treatment)	0.0519
- / IDV,RTV (before treatment)	0.0584
- / IDV,RTV (after treatment)	0.0720
- / RTV,SQV (before treatment)	0.0577
- / RTV,SQV (after treatment)	0.0687
- / IDV,RTV,SQV (before treatment)	0.0485
- / IDV,RTV,SQV (after treatment)	0.0681
IDV / IDV,NFV (before treatment)	0.0601
IDV / IDV,NFV (after treatment)	0.0690
IDV / IDV,RTV (before treatment)	0.0721
IDV / IDV,RTV (after treatment)	0.0787

Table S3. Estimates of molecular adaptation with *Hyphy*.

Dataset	*dN/dS*	95%CI down	95%CI up
- / - (before treatment)	0.456	0.456	0.457
- / - (after treatment)	0.445	0.445	0.445
- / APV (before treatment)	0.248	0.176	0.339
- / APV (after treatment)	0.315	0.236	0.410
- / ATV (before treatment)	0.222	0.172	0.281
- / ATV (after treatment)	0.254	0.201	0.315
- / IDV (before treatment)	0.301	0.263	0.342
- / IDV (after treatment)	0.379	0.340	0.422
- / LPV (before treatment)	0.264	0.222	0.312
- / LPV (after treatment)	0.269	0.227	0.315
- / NFV (before treatment)	0.235	0.217	0.253
- / NFV (after treatment)	0.268	0.250	0.286
- / RTV (before treatment)	0.242	0.190	0.303
- / RTV (after treatment)	0.302	0.246	0.367
- / SQV (before treatment)	0.250	0.201	0.306
- / SQV (after treatment)	0.268	0.220	0.322
- / IDV,RTV (before treatment)	0.233	0.163	0.322
- / IDV,RTV (after treatment)	0.244	0.178	0.325
- / RTV,SQV (before treatment)	0.206	0.147	0.279
- / RTV,SQV (after treatment)	0.271	0.204	0.351
- / IDV,RTV,SQV (before treatment)	0.264	0.184	0.363
- / IDV,RTV,SQV (after treatment)	0.529	0.412	0.666
IDV / IDV,NFV (before treatment)	0.402	0.310	0.510
IDV / IDV,NFV (after treatment)	0.495	0.395	0.611
IDV / IDV,RTV (before treatment)	0.329	0.261	0.409
IDV / IDV,RTV (after treatment)	0.442	0.360	0.536

Table S4. Number and position of positively selected sites and negatively selected sites. Only detected positively selected sites (PSSs) and negatively selected sites (NSSs) at 95% CI are shown. For each PSS the corresponding *p*-value is indicated after the parenthesis. "-" indicates naïve-treatment patients.

Scenario	DataSet	PSSs	NSSs
1	-	0	93:<br>1,2, 3, 4, 5, 6, 7, 8, 9, 11, 12, 13, 14, 15, 16, 17, 18, 20, 21, 22, 23, 24, 25, 26, 27, 28, 29, 30, 31, 32, 33, 34, 36, 38, 39, 40, 41, 43, 44, 45, 46, 47, 48, 49, 50, 51, 52, 53, 54, 55, 56, 57, 58, 59, 60, 61, 62, 64, 65, 66, 67, 68, 69, 70, 71, 72, 73, 74, 75, 76, 77, 78, 79, 80, 81, 82, 83, 84, 85, 86, 87, 88, 89, 90, 91, 92, 93, 94, 95, 96, 97, 98, 99
	-	0	92:<br>1,2, 3, 4, 5, 6, 7, 8, 9, 11, 12, 13, 14, 15, 16, 17, 18, 20, 21, 22, 23, 24, 25, 26, 27, 28, 29, 30, 31, 32, 33, 34, 36, 38, 39, 40, 41, 43, 44, 45, 46, 47, 48, 49, 50, 51, 52, 53, 54, 55, 56, 57, 58, 59, 60, 61, 62, 64, 65, 66, 67, 68, 69, 70, 71, 72, 73, 74, 75, 76, 78, 79, 80, 81, 82, 83, 84, 85, 86, 87, 88, 89, 90, 91, 92, 93, 94, 95, 96, 97, 98, 99
2	-	1:<br>63 (0)	26:<br>1, 3, 11, 17, 20, 22, 25, 27, 28, 31, 32, 34, 44, 47, 66, 67, 68, 70, 73, 74, 75, 79, 81, 83, 94, 98
	APV	2:<br>37 (0.00248), 63 (0.00002)	34:<br>11, 12, 20, 21, 22, 25, 27, 28, 29, 32, 40, 44, 45, 46, 49, 54, 56, 66, 68, 79, 80, 81, 82, 83, 85, 86, 87, 88, 91, 94, 95, 96, 98, 99
3	-	1:<br>37 (0.01383)	43:<br>7, 7, 9, 11, 16, 17, 20, 21, 22, 24, 25, 28, 30, 33, 34, 38, 39, 40, 43, 44, 46, 52, 58, 61, 65, 66, 68, 75, 76, 79, 80, 81, 83, 86, 87, 90, 91, 92, 94, 95, 96, 97, 99
	ATV	2:<br>63 (0.00404), 93 (0.03112)	36:<br>7, 9, 11, 17, 20, 21, 22, 24, 25, 28, 34, 38, 39, 44, 46, 52, 58, 61, 65, 66, 68, 75, 76, 79, 80, 81, 83, 86, 87, 90, 91, 92, 94, 95, 96, 99
4	-	5:<br>10 (0.00008), 35 (0.01782), 37 (0.00002), 63 (0), 93 (0.03455)	64:<br>1, 2, 3, 4, 5, 6, 7, 8, 9, 11, 16, 21, 22, 23, 24, 25, 26, 27, 29, 30, 32, 33, 38, 39, 40, 43, 45, 46, 48, 51, 52, 54, 58, 60, 61, 64, 65, 66, 67, 68, 73, 74, 75, 76, 78, 79, 80, 81, 82, 84, 85, 86, 87, 88, 90, 92, 94, 95, 96, 97, 98, 99
	IDV	7:<br>10 (0), 35 (0.01837), 37 (0.00024), 46 (0.04753), 63 (0.00198), 71 (0.04103), 93 (0.0121)	58:<br>1, 2, 3, 4, 5, 7, 8, 9, 11, 16, 17, 21, 22, 23, 25, 26, 27, 30, 32, 34, 38, 39, 40, 43, 45, 47, 48, 51, 52, 53, 55, 58, 59, 60, 61, 65, 66, 67, 68, 70, 74, 75, 78, 79, 80, 81, 83, 85, 86, 87, 88, 92, 94, 95, 96, 97, 98, 99
5	-	3:	54:

		10 (0.01485), 37 (0.01689), 63 (0.00001)	1, 2, 3, 4, 7, 9, 11, 14, 16, 17, 21, 22, 23, 24, 25, 27, 32, 38, 40, 43, 45, 48, 52, 54, 55, 56, 57, 58, 65, 66, 67, 68, 71, 72, 74, 75, 76, 78, 79, 80, 82, 83, 84, 86, 87, 88, 91, 92, 94, 95, 96, 97, 98, 99
	LPV	3:<br>10 (0.00749), 37 (0.02511), 63 (0.00017)	54:<br>1, 2, 3, 7, 9, 11, 14, 16, 17, 21, 22, 23, 24, 25, 27, 32, 38, 40, 46, 48, 51, 52, 54, 55, 56, 57, 58, 65, 66, 67, 68, 69, 71, 72, 74, 75, 76, 78, 79, 80, 83, 86, 87, 88, 90, 91, 92, 94, 95, 96, 97, 98, 99
6	-	3:<br>19 (0.04020), 37 (0), 63 (0)	86:<br>1,2, 3, 4, 5, 6, 7, 8, 9, 11, 13, 14, 15, 16, 17, 18, 20, 21, 22, 23, 24, 25, 26, 27, 28, 29, 30, 31, 32, 33, 34, 36, 38, 39, 40, 42, 43, 44, 45, 46, 48, 49, 50, 51, 52, 53, 54, 55, 56, 57, 58, 59, 60, 61, 65, 66, 67, 68, 69, 70, 71, 73, 74, 75, 76, 78, 79, 80, 81, 82, 83, 84, 85, 86, 87, 88, 89, 90, 91, 92, 94, 95, 96, 97, 98, 99
	NFV	4:<br>10 (0.01775), 19 (0.00295), 37 (0), 63 (0)	83:<br>1,2, 3, 4, 5, 6, 7, 8, 9, 11, 14, 15, 16, 17, 18, 20, 21, 22, 23, 24, 25, 26, 27, 28, 29, 30, 31, 32, 33, 34, 36, 38, 39, 40, 42, 43, 45, 46, 48, 49, 50, 51, 52, 53, 54, 55, 56, 58, 59, 60, 61, 65, 66, 67, 68, 70, 71, 73, 74, 75, 76, 78, 79, 80, 81, 82, 83, 84, 85, 86, 87, 88, 89, 90, 91, 92, 94, 95, 96, 97, 98, 99
7	-	1:<br>63 (0.00020)	39:<br>1, 2, 3, 6, 7, 11, 16, 17, 18, 22, 23, 27, 28, 33, 38, 40, 43, 45, 50, 52, 55, 56, 58, 61, 66, 68, 75, 78, 80, 81, 85, 87, 88, 91, 92, 94, 95, 96, 97
	RTV	1:<br>63 (0.00011)	40:<br>1, 2, 3, 7, 11, 14, 16, 17, 18, 22, 23, 24, 27, 28, 38, 40, 46, 48, 50, 52, 55, 56, 58, 61, 66, 68, 75, 76, 78, 80, 81, 85, 87, 88, 92, 94, 95, 96, 97, 99
8	-	1:<br>63 (0)	41:<br>2, 7, 9, 16, 17, 18, 20, 21, 23, 25, 27, 30, 33, 34, 40, 43, 44, 48, 52, 54, 55, 57, 58, 65, 66, 68, 70, 75, 76, 83, 86, 87, 88, 91, 92, 94, 95, 96, 97, 98, 99
	SQV	2:<br>35 (0.02464), 63 (0.00005)	43:<br>1, 2, 7, 9, 16, 17, 18, 20, 21, 23, 24, 25, 27, 30, 31, 33, 34, 40, 43, 44, 51, 52, 54, 55, 66, 68, 70, 75, 76, 79, 82, 83, 86, 87, 88, 91, 92, 94, 95, 96, 97, 98, 99
9	-	1:<br>63 (0,00397)	29:<br>4, 9, 14, 17, 21, 25, 29, 35, 37, 39, 40, 45, 48, 52, 54, 57, 66, 70, 72, 75, 80, 87, 88, 91, 94, 95, 96, 98, 99
	IDV, RTV	1:<br>63 (0,03740)	29:<br>1, 7, 17, 18, 21, 25, 29, 39, 40, 45, 48, 52, 57, 61, 66, 70, 72, 75, 79, 80, 83, 87, 88, 91, 92, 94, 96, 98, 99

10	-	1:<br>37 (0.00600)	28:<br>1, 3, 9, 14, 16, 17, 20, 27, 40, 47, 54, 56, 66, 67, 70, 74, 75, 76, 79, 82, 85, 86, 87, 88, 94, 95, 96, 98, 99
	RTV, SQV	2:<br>35 (0.04481), 37 (0.03160)	32:<br>1, 2, 3, 9, 14, 15, 16, 17, 18, 20, 27, 40, 56, 57, 61, 65, 66, 67, 68, 75, 79, 81, 83, 85, 87, 88, 91, 92, 94, 95, 96, 98, 99
11	-	1:<br>77 (0.01112)	12:<br>15, 22, 29, 40, 51, 60, 72, 74, 83, 84, 91, 94,
	IDV, RTV, SQV	6:<br>54 (0.01354), 63 (0.02167), 77 (0.00462), 82 (0.03294), 90 (0.03597), 93 (0.00623)	13:<br>15, 16, 17, 20, 22, 27, 45, 52, 67, 68, 83, 94, 99
12	IDV	3:<br>10 (0.00240), 63 (0.00946), 82 (0.04698)	20:<br>14, 17, 22, 25, 40, 45, 52, 66, 68, 75, 79, 80, 86, 87, 91, 94, 95, 96, 98, 99
	IDV, NFV	5:<br>10 (0.00068), 46 (0.00118), 62 (0.02108), 63 (0.00088), 90 (0.02295)	28:<br>1, 3, 4, 9, 14, 16, 17, 18, 22, 25, 28, 40, 45, 52, 55, 56, 58, 61, 68, 75, 79, 83, 86, 87, 94, 95, 96, 99
13	IDV	1:<br>10 (0.00026)	36:<br>1, 3, 4, 5, 6, 7, 8, 9, 11, 14, 17,, 21, 23, 25, 30, 32, 34, 40, 43, 48, 52, 68, 70, 73, 75, 78, 79, 80, 85, 86, 87, 92, 94, 95, 96, 99
	IDV, RTV	4:<br>10 (0.00026), 57 (0.04403), 72 (0.01765), 82 (0.03577)	30:<br>1, 2, 3, 4, 5, 6, 7, 9, 11, 17, 21, 23, 25, 26, 27, 30, 40, 43, 48, 52, 68, 70, 75, 80, 87, 92, 94, 95, 96, 99
